# Venous thromboembolism and severe COVID-19: a Mendelian randomization trial and transcriptomic analysis

**DOI:** 10.3389/fimmu.2024.1363598

**Published:** 2024-04-29

**Authors:** Liang Chen, Xiaoting Dai

**Affiliations:** ^1^ Department of Infectious Diseases, Taikang Xianlin Drum Tower Hospital, Affiliated Hospital of Medical College of Nanjing University, Nanjing, China; ^2^ Department of Infectious Diseases, Nanjing Lishui People’s Hospital, Zhongda Hospital Lishui Branch, Southeast University, Nanjing, China

**Keywords:** Mendelian randomization, severe COVID-19, venous thromboembolism, causal relationship, transcriptome analysis

## Abstract

**Introduction:**

Venous thromboembolism (VTE) is known to be intricately linked to severe COVID-19 (sCOVID-19) occurrence. Herein, we employed univariable Mendelian randomization (MR) and transcriptome analysis to predict the causal association and associated signaling networks between VTE and sCOVID-19.

**Methods:**

Potential VTE and sCOVID-19 association was assessed using MR-Egger, weighted median, simple mode, weighted mode, and inverse variance weighted (IVW) regression. We conducted independent univariable analyses involving VTE and sCOVID-19. Using heterogeneity, pleiotropy, and the Leave-One-Out examinations, we performed sensitivity analyses. Thereafter, we performed transcriptome analysis of the GSE164805 dataset to identify differentially expressed genes (DEGs) linked to single nucleotide polymorphisms (SNPs). Lastly, we conducted immune analyses.

**Results:**

Based on our univariable analysis, VTE was a strong indicator of sCOVID-19 development, and it was intricately linked to sCOVID-19. We further conducted sensitivity analysis to demonstrate the reliability of our results. Using differential analysis, we identified 15 major genes, namely, *ACSS2, CEP250, CYP4V2, DDB2, EIF6, GBGT1, GSS, MADD, MAPK8IP1, MMP24, YBPC3, NT5DC3, PROCR, SURF6*, and *YIPF2*, which were strongly connected to suppressive adaptive immune as well as augmented inflammatory cells. In addition, we uncovered strong associations with most differential immunologic gene sets, such as, the Major Histocompatibility Complex (MHC), immunoactivators, and immunosuppressors.

**Conclusion:**

Herein, we demonstrated we strong association between VTE and enhanced sCOVID-19 risk. We also identified 15 DEGs which potentially contribute to the shared immunologic pathogenesis between VTE and sCOVID-19.

## Introduction

Coronavirus disease 2019 (COVID-19) is a continuing global hazard with a total incidence of > 70 million cases and total mortality exceeding 6,900,000 deaths as of June, 2023, according to WHO (1). Venous thromboembolism (VTE) is a frequently occurring comorbidity of COVID-19, with predicted cases reaching 25-30% (2). VTE impacts multiple organs, namely, vasculature of the lungs, legs, spleen, heart, and brain. Despite anticoagulant prophylaxis administrated, VTE rates can be as much as 25-69% among critically ill COVID-19 patients in the intensive care unit (ICU), and 7% among general medical floor patients (2). Asymptomatic deep vein thrombosis (DVT) is approximately 85% among critically ill patients and 46% among hospitalized patients who undergo ultrasound screening (3). One meta-analysis involving 86 research articles suggested a VTE incidence of 7.9% among non-ICU and 22.7% among ICU COVID-19 patients, along with pulmonary embolism occurrences of 3.5% and 13.7%, respectively (4). These comorbidities are intricately linked to multiorgan failure and enhanced mortality rates, particularly among patients with advanced disease. Patients exhibiting augmented thrombotic manifestations (e.g., elevating D-dimer levels, DVT, pulmonary embolism, ischemic stroke, et al) are more susceptible to developing severe COVID-19 (sCOVID-19) (5–7). Hence, thrombosis-related complications are a critical indicator of mortality among COVID-19 patients (2).

Notably, the causal relationship between VTE and sCOVID-19, and the regulation of sCOVID-19 by which genes have not been reported. Marked inherent defects exists in conventional research, hence most research are not able to completely eliminate reverse causality and confounding factors possibilities, thereby potentially introducing bias in acquired conclusion. Additionally, randomized controlled studies (RCTs) often have ethical and impractical concerns owing to the need for extensive personnel resources and prolonged follow-up. Mendelian randomization (MR) is commonly employed for detection of causal associations between hazard factors and disease outcomes (8). It utilizes environmental exposure-associated genetic variations (termed as instrumental variables [IVs]) to evaluate the relationship between various exposures (such as, VTE) and outcomes (namely, sCOVID-19). Given that the genetic variants are arbitrarily designated at conception before disease onset, MR analysis can effectively exclude confounding factors while screening for causal relationship for a particular outcome (9).

VTE and COVID-19 may share a common genetic architecture, which has not been clarified. To fill this gap, we employed VTE as the exposure factor, single-nucleotide polymorphisms (SNP) as the IV, and sCOVID-19 with respiratory failure as the outcome genetically estimate the causal relationship between VTE and sCOVID-19 using MR analysis. Additionally, we conducted transcriptomic analysis to identify differentially expressed genes (DEGs) and associated immunopathologic profiles. Our findings highlight novel pathways of VTE and sCOVID-19 association, and provide theoretical supports for the in-depth understanding of sCOVID-19.

## Materials and methods

### Data acquisition

We retrieved traitID and GWAS data for venous thromboembolism (GWAS ID: finn-b-I9_VTE) and sCOVID-19 with respiratory failure (GWAS ID: ebi-a-GCST90000255) from the IEU OpenGWAS database. In addition, we obtained 27 sCOVID-19 (18 males and 9 females, with median age of 50 yrs.) and 24 healthy control (17 males and 9 females, with median age of 47 yrs.) PBMC samples from the GSE164805 dataset, which was acquired from the Gene Expression Omnibus (GEO; https://www.ncbi.nlm.nih.gov/geo/).

### Data pre-processing

Exposure factors reading and filtering employed the extract_instruments function of the TwoSampleMR package (10) (p < 5×10^-8^). To conduct linkage disequilibrium analysis (LDA), we clumped instruments, which endured independence (r^2 = ^0.001 and kb = 10000). Lastly, we eliminated IVs with strong outcome association to minimize confounding effects using PhenoScanner.

### MR univariable and sensitivity analyses

Following IV filtration, we acquired input information for MR univariable analysis. Using the Harmonise_data function (TwoSampleMR package), we harmonized the effect equivalents and sizes. The primary MR procedure utilized MR-Egger (11), weighted median (12), Simple mode, weighted mode (13) and inverse variance weighted (IVW) regression (14) analyses. The study outcomes are provided as scatter, forest, and funnel plots. Additionally, we employed heterogeneity (mr_ heterogeneity _test function), pleiotropy (mr_pleiotropy_test function and MRPRESSO package (https://github.com/rondolab/MR-PRESSO)), and the Leave-One-Out (LOO) sensitivity tests to assess dependability of the sensitivity analysis results. Leave-one-out analysis can reveal that the overall estimates are not disproportionately affected by any individual SNP. The aforementioned assessments were independently performed between the exposure factor and sCOVID-19.

### DEG analysis

DEG expression analysis utilized gene expression profiles from the GSE164805 dataset as well as the eQTLGen database for SNP’s cis-expression quantitative trait loci (cis-eQTL) related genes. Subsequently, we assessed alterations in gene expression between sCOVID-19 and Control samples, and present acquired results via violin plots (p < 0.05). DEGs are described as major genes in the following analyses.

### Gene set enrichment analysis

We utilized the ‘clusterProfiler’ program in GSEA, as well as ‘c2.cp.kegg.v7.5.1.symbols.gmt’ of the MSigDB database, to examine potential KEGG networks associated with major genes. Significance was determined at p.value < 0.05.

### Immune invasion analysis

Using the single-sample gene set enrichment analysis (ssGSEA) algorithm (GSVA package), we computed the relative profiles of 28 immune cells and scores of 13 immune invasion-related networks in the GSE164805 dataset. Alterations in immune invasive cells and associated networks between the sCOVID-19 and healthy control samples were evaluated using the Wilcoxon test. Spearman correlation analysis was conducted between major genes and differential immune invasive cells and signaling networks.

### Immune profile analysis

We retrieved data pertaining to the immunopathological profile, including Major Histocompatibility Complex (MHC), immunoactivator, and immunosuppressor from the TISIDB (http://cis.hku.hk/TISIDB/index.php) database. Spearman correlation analysis was conducted between major genes and differential immunologic profile-related gene sets.

### Statistical analysis

Significance was determined at p.adjust value < 0.05. Marked alterations between cohorts were assessed using the Wilcox test.

## Results

### Relationship between VTE and sCOVID-19

**Figure 1:**
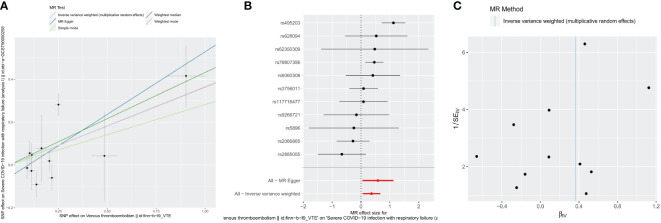
Causal effects for VTE on the risk of sCOVID-19 by MR analyses. **(A)** Scatter plots of MR analyses. **(B)** forest plots of MR analyses. IVW method demonstrated a causal association between VTE and sCOVID-19. **(C)** funnel plots of heterogeneity test. The left-right symmetrical in funnel plot indicated that the result was unlikely to be affected by potential bias, and there was no heterogeneity between these SNPs in the causal relationship.

Following strict screening, 11 SNPs were identified as IV. Using the IVW method, we demonstrated a causal association between VTE and sCOVID-19 (p = 0.018338555) (**Table 1**). Results from 5 algorithms were plotted as scatter plots, and all acquired data closely corroborated with prior results (**Figure 1A**). VTE was thus determined as a hazard factor for sCOVID-19. To elucidate the diagnostic efficiency of individual SNP loci for exposure factors estimation of patient outcomes, we generated forest plots. The lateral left SNP point confirmed that the locus was a safety factor, whereas the lateral right SNP point was a hazard factor. Similar to prior outcomes, VTE, in this report, was also demonstrated as a robust hazard factor for sCOVID-19 (**Figure 1B**). Lastly, based on the randomness judgement, the factor sample distributions was left-right symmetrical in funnel plot, which indicated that the above result was unlikely to be affected by potential bias. Meanwhile, it also showed that there was no heterogeneity in the association and the result strongly corroborated with the Mendel’s second law random grouping (**Figure 1C**).

**Table 1:** The results of Mendelian randomization analyses.

Exposure	Method	nsnp	β	SE	P val	OR	OR_95% CI_ low	OR_95% CI_ up
VTE ||id:finn-b-I9_VTE	MR Egger	11	0.585558505	0.272178877	0.059902593	1.795993776	1.053468345	3.061879989
VTE ||id:finn-b-I9_VTE	Weighted median	11	0.431889411	0.14180577	0.002321852	1.540164781	1.166432683	2.033642908
VTE ||id:finn-b-I9_VTE	IVM	11	0.364977721	0.154736123	0.018338555	1.440481915	1.0636378	1.950840924
VTE ||id:finn-b-I9_VTE	Simple mode	11	0.281950247	0.301222764	0.371316709	1.32571276	0.734587346	2.392519189
VTE ||id:finn-b-I9_VTE	Weighted mode	11	0.362861528	0.203350057	0.10467283	1.437436801	0.964925307	2.141331089

VTE, venous thromboembolism; IVM, inverse variance weighted model; SNP, single nucleotide polymorphisms; SE, standard error; OR, odd ratio; CI, confidence interval.

### Sensitivity analysis

**Figure 2:**
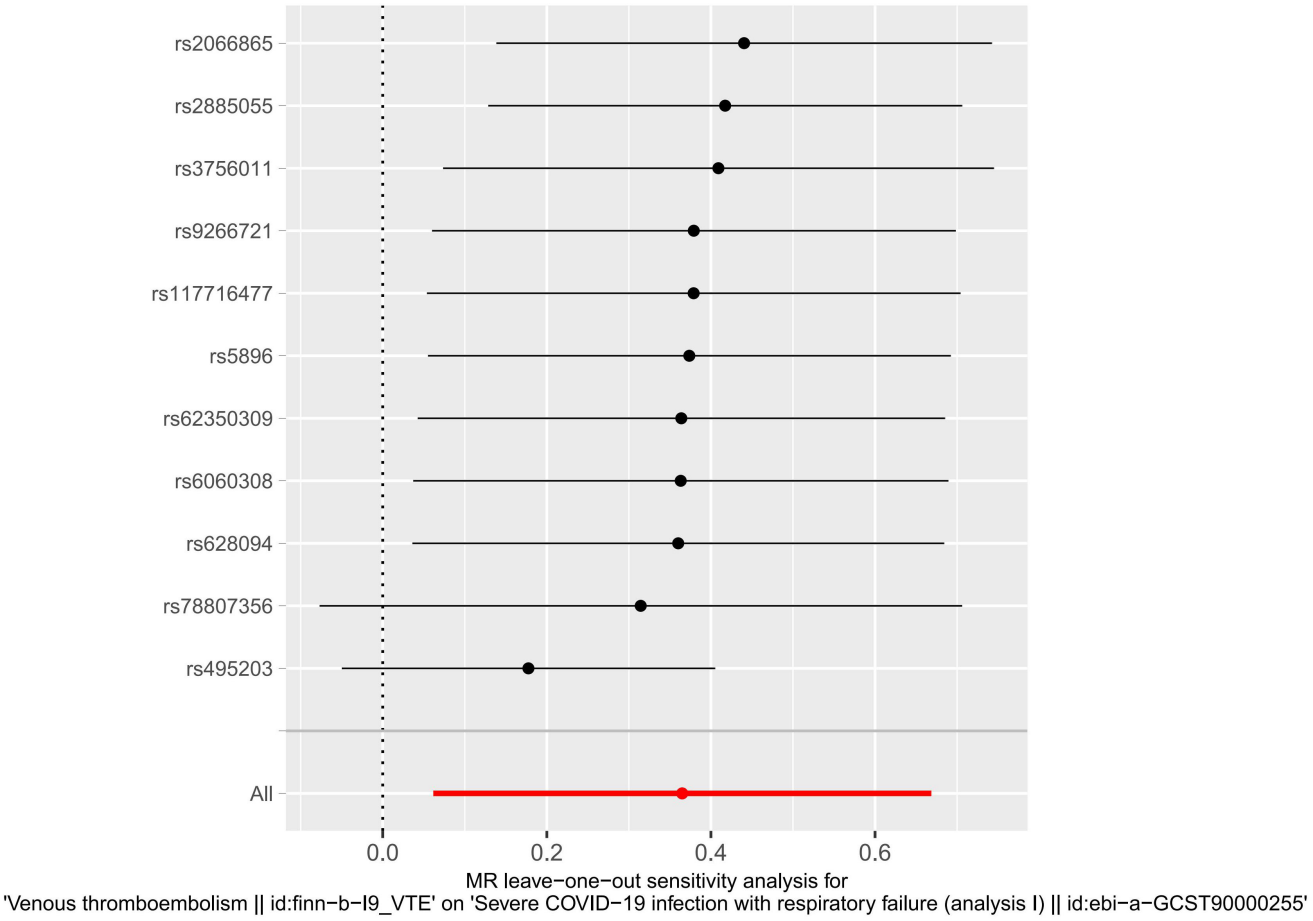
MR leave−one−out sensitivity analysis for VTE on sCOVID-19. LOO analysis revealed that the results were similar to IVW analysis, suggesting strong data reliability.

Heterogeneity was determined at p value was < 0.05. Herein, the VTE p value was 0.002233986, and heterogeneity was detected among the SNPs (**Table 2**). Although there was heterogeneity between the two disease populations, we would not consider it because the MR Analysis results described above were significant. The pleiotropy test results suggested no presence of horizontal SNPs multi-effect (**Table 2**). Additionally, the LOO analysis revealed that the results were similar to IVW analysis, suggesting strong data reliability (**Figure 2**). Taken together, these aforementioned results indicated that VTE strongly contributes to sCOVID-19.

**Table 2:** The results of heterogeneity and pleiotropy test.

Exposure	Outcome	Method	Heterogeneity test			Pleiotropy test		
			Q	Q_df	Q_pval	egger_intercept	se	Q_pval
VTE || id:finn-b-I9_VTE	sCOVID-19 || id:ebi-a-GCST90000255	MR Egger	24.74791701	9	0.003263059	-0.003774479	0.022041057	0.86828126
		IVM	27.42001115	10	0.002233986			

VTE, venous thromboembolism; sCOVID-19, severe COVID-19; IVM, inverse variance weighted model; se, standard error.

### Identification and functional enrichment analysis of major sCOVID-19-related genes

**Figure 3:**
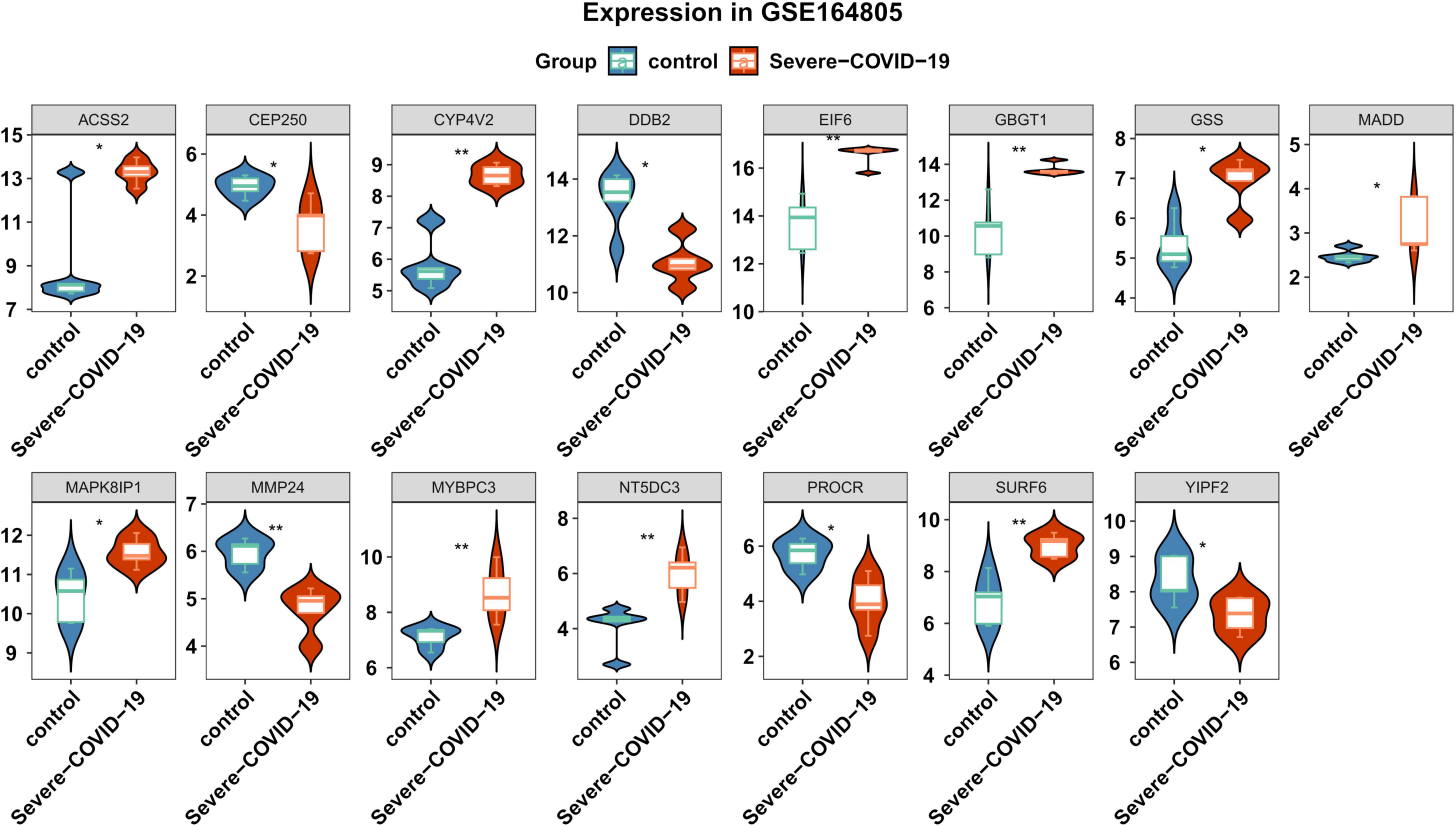
Expression levels of the genes associated with SNPs between sCOVID-19 and health control groups. 15 DEGs were identified between sCOVID-19 and healthy controls.

**Figure 4:**
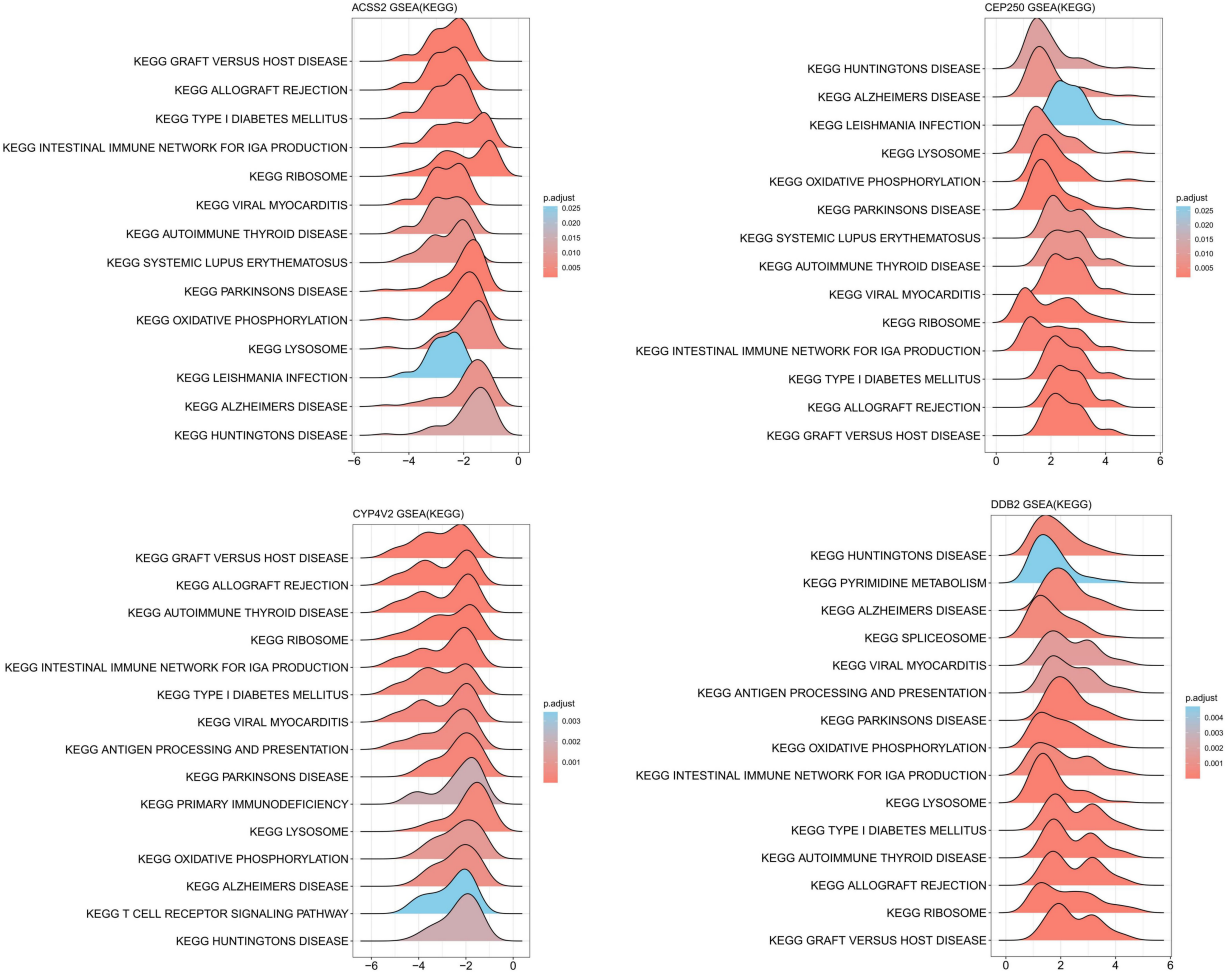
KEGG-ssGSEA results of ACSS2, CEP250, CYP4V2 and DDB2 gene. The DEGs were strongly enriched in immune- or inflammation response-associated networks.

We conducted differential analysis to elucidate alterations in SNP-related genetic profiles between sCOVID-19 and normal samples. In total, we identified 37 SNP-related genes within the GSE145926 dataset. Among these, there were 15 DEGs between sCOVID-19 and healthy controls, and these included 10 highly expressed (*ACSS2, CYP4V2, EIF6, GBGT1, GSS, MADD, MAPK8IP1, MYBPC3, NT5DC3* and *SURF6*), and 5 scarcely expressed genes (*CEP250*, *DDB2*, *MMP24*, *PROCR* and *YIPF2*) in sCOVID-19 (**Figure 3**). To further elucidate potential physiological functions of these DEGs in sCOVID-19, we conducted single-gene GSEA. Based on our KEGG analysis, the DEGs were strongly enriched in immune- or inflammation response-associated networks, indicating that these networks were critical for sCOVID-19 pathogenesis (**Figure 4**, **Supplementary Figure 1**).

### Assessing DEG function in the sCOVID-19 immune microenvironment

**Figure 5:**
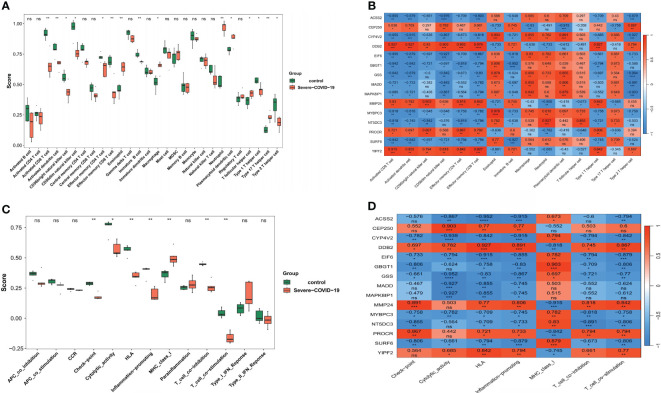
Analysis of the role of key genes in sCOVID-19 immune microenvironment. **(A)** The expression abundance of 28 types of immune cells in sCOVID-19 and health control groups. 15 immune cell abundances that were considerably different in sCOVID-19 versus control samples. **(B)** The heatmap of the correlation between the key genes and immune cells. Marked reductions in the adaptive immune response cells, and considerable elevations in the inflammatory and myeloid cells were observed in sCOVID-19 samples. **(C)** The scores of 13 immune pathways in sCOVID-19 and health control groups. Marked differences in the scores of 7 immune-related networks (check-point, cytolytic_activity, HLA, inflammation-promoting, MHC_class_I, T_cell_co-inhibition, and T_cell_co-stimulation) were detected. **(D)** The heatmap of the correlation between the key genes and immune pathways. Five scarcely expressed DEGs were positively linked to check-point, cytolytic_activity and T_cell_co-inhibition networks. Alternately, the 10 highly expressed DEGs were directly associated with the MHC_class_I and T_cell_co-stimulatory networks. *: p < 0.05, **:p < 0.01, ns: not significant.

Given strong potential associations between sCOVID-19 pathophysiology and the IME, we next explored the IME of sCOVID-19 patients by examining the expression profiles of 28 immune-related cells. Interestingly, we demonstrated 15 immune cell abundances that were considerably different in sCOVID-19 versus control samples (**Figure 5A**). Among sCOVID-19 samples, we revealed marked reductions in the adaptive immune response cells (e.g., activated CD8 T cell, activated dendritic cell, natural killer cell, effector memory CD4 T cell, effector memory CD8 T, immature B cell, and so on), along with considerable elevations in the inflammatory and myeloid cells (e.g., neutrophil, eosinophil and macrophage, etc.) in sCOVID-19 versus normal samples. We next demonstrated strong associations between major genes and differential immune cells. Particularly, 5 scarcely expressed DEGs (*CEP250*, *DDB2*, *MMP24*, *PROCR* and *YIPF2*) were directly correlated with adaptive immune cells. Conversely, 10 highly expressed genes (*ACSS2, CYP4V2, EIF6, GBGT1, GSS, MADD, MAPK8IP1, MYBPC3, NT5DC3* and *SURF6*) were directly associated with inflammatory and myeloid cells (**Figure 5B**). Additionally, we demonstrated marked differences in the scores of 7 immune-related networks, namely, check-point, cytolytic_activity, HLA, inflammation-promoting, MHC_class_I, T_cell_co-inhibition, and T_cell_co-stimulation (**Figure 5C**). Based on our analysis, the 5 scarcely expressed DEGs were positively linked to check-point, cytolytic_activity and T_cell_co-inhibition networks. Alternately, the 10 highly expressed DEGs were directly associated with the MHC_class_I and T_cell_co-stimulatory networks (**Figure 5D**). Together, these findings indicated that the DEGs are essential for the sCOVID-19 IME.

### DEGs were intricately linked to the immunopathological profiles

**Figure 6:**
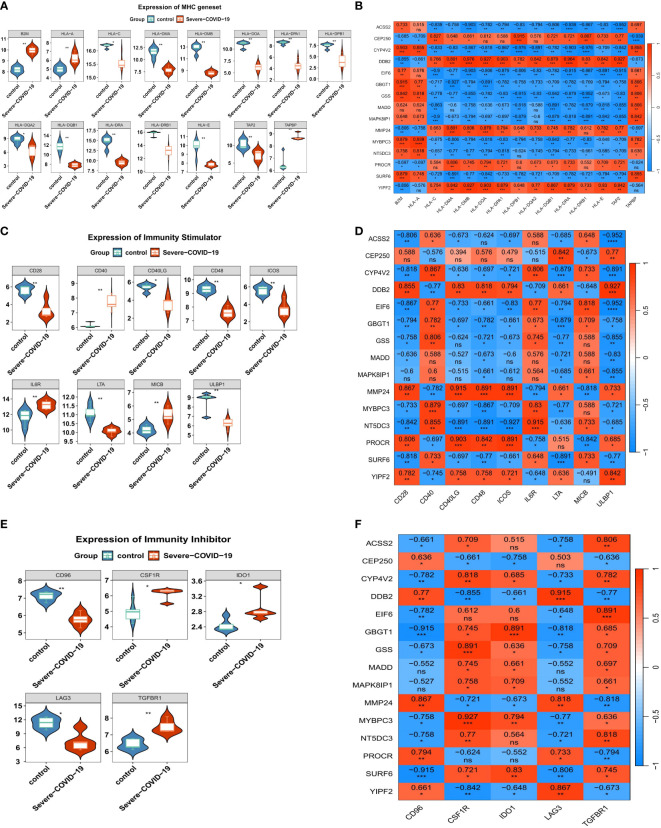
The immune characteristics associated with the key genes. **(A)** The expression levels of MHC genes in sCOVID-19 and health control groups. In terms of the MHC gene sets, we demonstrated 3 DEGs (B2M, HLA-A and TAPBP) that were strongly up-regulated in sCOVID-19 versus control samples. In contrast, 12 DEGs (HLA−C, HLA−DMA, HLA−DMB, HLA−DOA, HLA−DPA1, HLA−DPB1, HLA−DQA2, HLA−DQB1, HLA−DRA, HLA−DRB1, HLA−E and TAP2) revealed the opposite trend. **(B)** The heatmap of the correlation between the key genes and MHC genes. B2M, HLA-A and TAPBP genes were inversely related to the 5 strongly diminished DEGs (CEP250, DDB2, MMP24, PROCR and YIPF2). **(C)** The expression levels of immunoactivator genes in sCOVID-19 and health control groups. CD40, IL6R and MICB were strongly upregulated in the sCOVID-19 versus normal samples. In contrast, the CD28, CD40LG, CD48, ICOS, ULBP1 and LTA contents exhibited the opposite response. **(D)** The heatmap of the correlation between the key genes and the immunoactivator. CEP250, DDB2, MMP24, PROCR and YIPF2 contents were directly related to the majority down-regulated immunoactivators. **(E)** The expression levels of immunosuppressor genes in sCOVID-19 and health control groups. CSF1R, IDO1 and TGFBR1 contents were substantially elevated in the sCOVID-19 versus normal samples. Alternately, the CD96 and LAG3 levels revealed the opposite trend. **(F)** The heatmap of the correlation between the key genes and the immunity inhibitors. CEP250, DDB2, MMP24, PROCR and YIPF2 contents were directly related to the CD96 and LAG3 levels.

To further examine the associations between DEGs and sCOVID-19 immunopathological profiles, we performed the following analyses. In terms of the MHC gene sets, we demonstrated 3 DEGs (*B2M, HLA-A* and *TAPBP*) that were strongly up-regulated in sCOVID-19 versus control samples. In contrast, 12 DEGs (*HLA−C, HLA−DMA, HLA−DMB, HLA−DOA, HLA−DPA1, HLA−DPB1, HLA−DQA2, HLA−DQB1, HLA−DRA, HLA−DRB1, HLA−E* and *TAP2*) revealed the opposite trend (**Figure 6A**). We also demonstrated that the *B2M, HLA-A* and *TAPBP* genes were inversely related to the 5 strongly diminished DEGs (*CEP250, DDB2, MMP24, PROCR* and *YIPF2*) (**Figure 6B**). In case of immunoactivators, *CD40, IL6R* and *MICB* were strongly upregulated in the sCOVID-19 versus normal samples. In contrast, the *CD28, CD40LG, CD48, ICOS, ULBP1* and *LTA* contents exhibited the opposite response (**Figure 6C**). Furthermore, we revealed that the *CEP250, DDB2, MMP24, PROCR* and *YIPF2* contents were directly related to the majority down-regulated immunoactivators (**Figure 6D**). Lastly, in terms of immunosuppressors, the *CSF1R, IDO1* and *TGFBR1* contents were substantially elevated in the sCOVID-19 versus normal samples. Alternately, the *CD96* and *LAG3* levels revealed the opposite trend (**Figure 6E**). Moreover, the *CEP250, DDB2, MMP24, PROCR* and *YIPF2* contents were directly related to the *CD96* and *LAG3* levels (**Figure 6F**).

## Discussion

Some observational investigations suggested a strong association between abnormal coagulation among COVID-19 patients and enhanced mortality risk. Moreover, certain epidemiological research indicated close correlation between VTE and other factors (8, 9). MR utilizes genetic investigation and inter-trait causal estimates to determine associations between specific variables. Genetic studies are typically devoid of data from environmental cues. This differentiates MR from epidemiological studies, and provides it with its unique advantage. Herein, we analyzed the shared genetic etiology between VTE and sCOVID-19 using the latest large-scale data. We demonstrated a marked positive causal association between VTE and sCOVID-19 risk. These findings contributed to a better understanding of the role of VTE in sCOVID-19 and had important clinical implications. Thrombotic events that occur frequently in sCOVID-19 are primarily VTE and are associated with increased disease severity and worsening clinical outcomes.

Some retrospective studies inferred that VTE rates in patients with sCOVID-19 appear to be in the higher range compared to patients with other diseases in the intensive care unit (ICU) (15). In our finding, VTE was a risk factor for sCOVID-19 patients. On average, sCOVID-19 patients experience enhanced thrombotic event risk, along with substantially severe disease progression, relative to patients who suffer from respiratory infections of other causes (2–4, 8). Excess inflammatory response, marked with augmented cytokine and chemokine release is strongly connected to adverse clinical outcomes (1–4, 16, 17). Immunothrombosis that results from severe inflammation occurs due to endothelial dysfunction, platelet hyperreactivity, and coagulation activation, which significantly enhances thrombosis risk in the micro- and macrovascular bed (17). These thrombotic events strongly influence the lung micro- and marcrovasculature thereby promoting respiratory symptoms, which eventually give rise to severe acute respiratory distress syndrome (16). Emerging evidences suggested that neutrophils, immunogenic platelets, and impaired coagulation cascade synergistically enhance immunothrombotic tissue injury among sCOVID-19 patients, as was evidenced within autopsy specimens and circulation of sCOVID-19 patients (18). Discreet neutrophil and platelet activation states, as well as enhanced plasmatic coagulation among sCOVID-19 patients heavily contribute to disease progression. Hence, immunothrombosis plays a central pathogenic role in sCOVID-19 connecting it to respiratory failure and systemic hypercoagulability. Prior cross-trait meta- and colocalization analyses indicated that certain known VTE-associated genes (e.g., *ABO, FUT2*, and *ADAMST13*, et al) were strongly associated with COVID-19 diseases severity (19, 20). An MR investigation involving 12 coagulation factors and COVID-19 severity reported that VWF is strongly correlated with COVID-19 susceptibility and hospitalization (19). Huang and colleagues performed an MR analysis to genetically predict that VTE is intricately linked to enhanced COVID-19 infection and hospitalization risks (20). This report further validated the direct correlation between genetically estimated VTE and enhanced sCOVID-19 with respiratory failure risk.

Immuno- and inflammatory responses critically modulate sCOVID-19 pathogenesis (21–23). Similar to multiple reported observational investigations, we revealed that sCOVID-19 patients experience remarkably suppressive adaptive immune responses, which are typically manifested by a sustained downregulation of serum T cell quantity (particularly, CD8+T cells) and DC, as well as an enhanced quantity of myeloid and inflammatory cells. Prior investigations indicated that the neutrophil/CD8+T cell ratio is a strong indicator of COVID-19 severity. At present, there are 2 broad hypotheses, e.g., inability to generate a timely antiviral response and inability to manage SARS-CoV-2-induced inflammatory responses, that potentially explain sCOVID-19 pathophysiology (22). Timely type I IFN synthesis by host cells is crucial for managing viral replication and disease promotion. Impaired type I IFN response diminishes DC numbers, and causes severe T cell lymphopenia (22). Till date, there is no consensus on the pathology of lymphopenia. However, possible reasons include intense lymphocyte recruitment to infected organs, apoptotic mechanisms using the Fas/Fas or TNF-related ligand, and corticosteroids usage to relieve inflammation (23). Excessive serum myeloid progenitor contents, particularly, neutrophilia, along with rising macrophages, monocytes and eosinophil levels, are strong inducers of severe disease, and they are often triggered during the early phase of infection, likely due to delayed viral clearance (21). Secondary bacterial infections that occur during the immunosuppression stage that follows the hyperinflammation phase also result in neutrophilia (22). Serum myeloid cells synthesis large quantities of inflammatory molecules that enhance vascular permeability and inflict organ damage. Patients with sCOVID-19 experience augmented neutrophil extracellular traps (NETs), which are regions of DNA material integrated with neutrophil-secreted antimicrobials and oxidant enzymes for infection management. NETs accelerate lung injury and immunothrombosis among COVID-19 patients (21).

Herein, we screened 15 DEGs that have close correlation with VTE and sCOVID-19. The human *EPCR* gene (*PROCR*), residing on chromosome 20 at position q11.2, spans 8kb, and consists of 4 exons (24). *PROCR* encodes the endothelial protein C receptor (EPCR), which abrogates blood coagulation (24, 25). Thrombomodulin interacts with thrombin to alter the protein from its original form to a procoagulant enzyme to an activator of protein C (PROC-encoded) (25). This process is accelerated by protein C association with EPCR. Upon interaction with EPCR via protease-driven receptor-1 cleavage on endothelial cells, activated PC, in combination with its cofactor protein S, suppresses thrombin production through inactivation factors VIIIa and Va to generate a cytoprotective/anti-inflammatory environment. Pulmonary endothelial cells belonging to sCOVID-19 patients exhibiting coagulation abnormalities revealed diminished anticoagulant thrombomodulin and EPCR expressions, indicating decreased lung endothelial ability to reduce thrombin propagation (26). Apart from its involvement in coagulation, EPCR also modulates both immune- and inflammatory responses (27–29). In recent investigations, EPCR was recognized as a TCR ligand for a Vδ2−γδ T cell subpopulation that elicits a marked rise in T cell quantity in response to infection (28). EPCR serves as a co-inhibitory receptor on the T cell surface. Procr+ CD8+T cells, unlike Procr-CD8+ T cells, typically exhibit an exhausted phenotype, whereby it generates minimal TNF-α and IL-2 contents while simultaneously augmenting IL-10 levels (27, 29). The Procr network stimulates a severely exhausted phenotype among CD8+ T cells. In chicken models, CD1 and EPCR constitute a discreet class-I-like gene subfamily that predates mammalian emergence (30). Similar to earlier conclusions, in this report, we revealed that *PROCR* positively regulates cytolytic_activity and T_cell_co-inhibition networks in sCOVID-19.

Herein, we demonstrated that *PROCR* not only directly regulates adaptive immune cells, but also modulates myeloid and inflammatory cells. Prior investigations revealed that EPCR interacting with coagulation proteases (namely, thrombin or tissue factor), or anticoagulant proteins (namely, activated protein C) to specific cell receptors on mononuclear or endothelial cells may influence cytokine synthesis (e.g., TNF-a, IL-1, IL-6, and IL-8, etc.) or inflammatory cell apoptosis (27, 29–31). Furthermore, EPCR itself possesses anti-inflammatory activities. EPCR suppression mediated by targeted monoclonal antibody further aggravates both coagulatory and inflammatory responses to *Escherichia coli* infusion (32). Of note, the EPCR (sEPCR) soluble form alters its function towards coagulatory and inflammatory responses following endothelial injury. Nergiz Bayrakcia et al. reported that sCOVID-19 patients with ground-glass opacity and bilateral involvement on thorax CT often possess enhanced circulating sEPCR contents (33). Therefore, EPCR/*PROCR* regulates crosstalk between coagulation and immune/inflammatory responses.

CEP250, otherwise called C-NAP1, modulates centrosome cohesion, centriole biogenesis, and centrosome duplication at distinct cellular cycles (34). CEP family proteins critically maintain the checkpoint signal mechanism, along with centriole biogenesis. Gordon et al. recently introduced CEP250 as candidate therapeutic targets in COVID-19, and revealed strong association between CEP250 and the SARS-CoV-2 nonstructural protein-13 (Nsp13) (35). Host Nsp13 (golgins) interactions facilitate drastic Golgi trafficking reconfigurations. During disease onset, ACE2 regulates virus entry into human cells, however, during disease progression, CEP250 proteins serve a more significant role. DDB2 is known to control nucleotide excision repair (NER), a major DNA repair system (36). It was revealed that the DDB2 expression in serum leukocytes was significantly downregulated following COVID-19 infection (37). MMP-24 is a member of the matrix metalloproteinases family, and they are zinc-dependent extracellular matrix (ECM) remodeling endopeptidases that can destroy almost all ECM (38). MMP-24 regulates angiogenesis, cell repair, and tissue remodeling. Henrik et al. demonstrated in inflammatory central nervous system mice models that the *MMP-24* expression was drastically diminished, which, in turn, was linked to enhanced cytokine (e.g., TNF-α, IFNγ and IL-1β) and lymphocytes invasion (39). In traumatic DVT rat model, MMP-24 is scarcely expressed during thrombus resolution, indicating a potential role in anti-fibrinolysis and thrombolysis regulation (40). However, till date, there are very limited studies that investigated the relevant genes involved in VTE and severe COVID-19 pathologies.

This research encountered several limitations. Firstly, despite utilizing the largest COVID-19 GWAS summary statistics till date, the available data was based on meta-analysis of numerous relevant studies, thus, the samples may be heterogeneous. For instance, the baseline clinical profiles, demographics, comorbid disease distributions, and so on may have differed across cohorts. The control population were unscreened. Therefore, there is a potential that asymptomatic or patients with mild symptoms may have been included as healthy controls. Secondly, since MR analyses extrapolate causal hypotheses via exploitation of arbitrary genet variant allocation, it was challenging to use MR to delineate between mediation and pleiotropy. Therefore, the many variants in our samples may influence ≥1 phenotypes. Thirdly, this research lacked supplemental mediator and observational analyses which would further validate the causal link between VTE and COVID-19. Finally, owing to an European ancestral-rich subject population, our conclusions may be inapplicable towards people of other ethnicities, lifestyles, and cultural backgrounds.

## Conclusions

Herein, we performed a univariable MR analysis to determine the causal relationship between VTE and sCOVID-19 risk. We demonstrated that VTE is intricately linked to sCOVID-19 occurrence. Additionally, we identified 15 DEGs involved in the immunologic mechanisms of VTE that drive sCOVID-19 pathogenesis. These genes provide new insight into the associated signaling network behind VTE and sCOVID-19, and may be utilized as new therapeutic targets for the treatment and prevention of sCOVID-19.

## Data availability statement

The original contributions presented in the study are included in the article/**Supplementary Material**. Further inquiries can be directed to the corresponding author.

## Author contributions

LC: Conceptualization, Data curation, Formal analysis, Funding acquisition, Investigation, Methodology, Project administration, Resources, Software, Supervision, Validation, Visualization, Writing – original draft, Writing – review & editing. XD: Investigation, Supervision, Validation, Visualization, Writing – original draft, Writing – review & editing.
