# Supplementary material for: Venous thromboembolism and severe COVID-19: a Mendelian randomization trial and transcriptomic analysis
Source: Front Immunol. 2024 Apr 29;15:1363598. doi: 10.3389/fimmu.2024.1363598 (PMC11089160; doi:10.3389/fimmu.2024.1363598)
Supplement: Supplementary file 1 [file DataSheet_1.pdf]

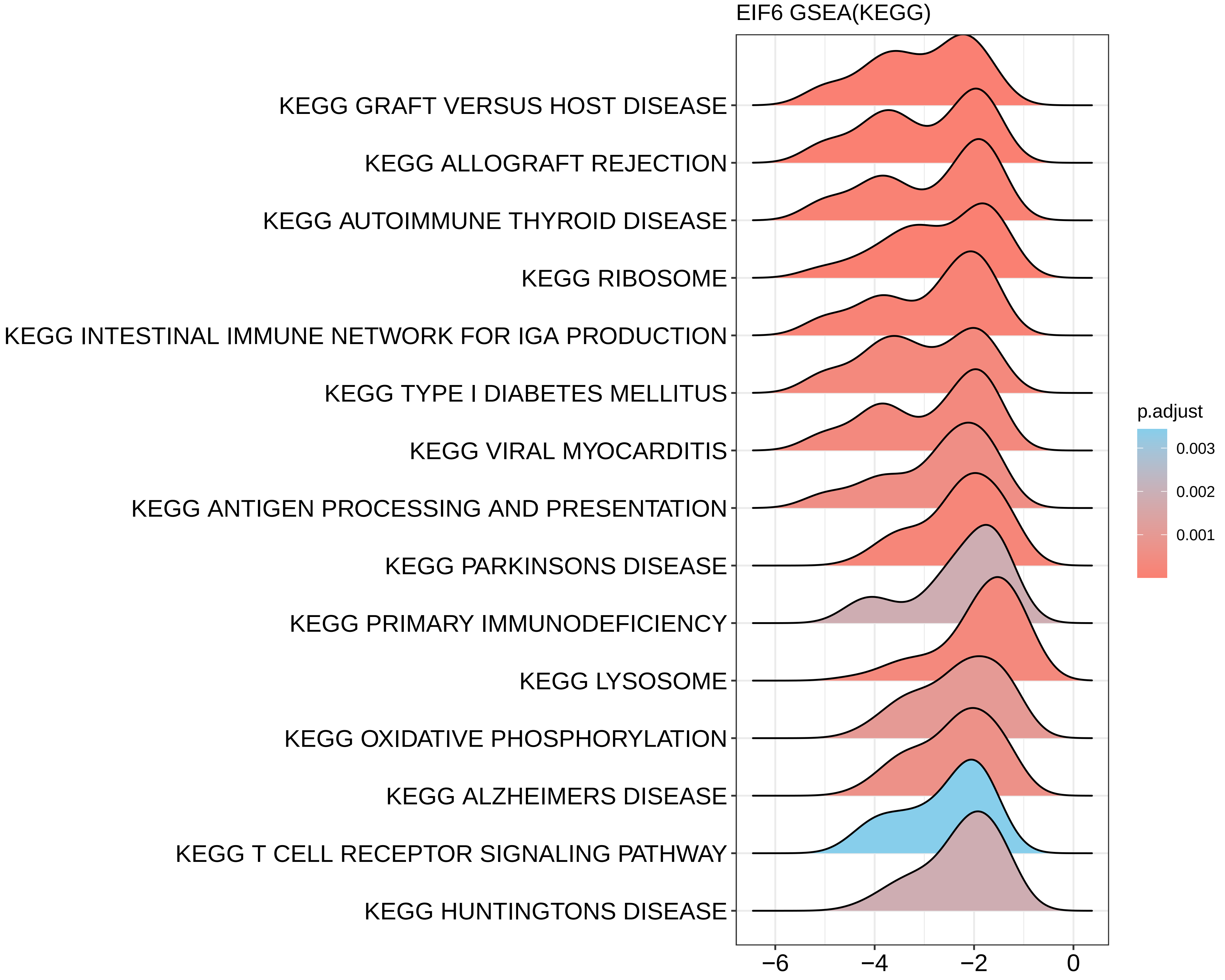

EIF6 GSEA(KEGG)
KEGG GRAFT VERSUS HOST DISEASE
KEGG ALLOGRAFT REJECTION
KEGG AUTOIMMUNE THYROID DISEASE
KEGG RIBOSOME
KEGG INTESTINAL IMMUNE NETWORK FOR IGA PRODUCTION
KEGG TYPE I DIABETES MELLITUS
KEGG VIRAL MYOCARDITIS
KEGG ANTIGEN PROCESSING AND PRESENTATION
KEGG PARKINSONS DISEASE
KEGG PRIMARY IMMUNODEFICIENCY
KEGG LYSOSOME
KEGG OXIDATIVE PHOSPHORYLATION
KEGG ALZHEIMERS DISEASE
KEGG T CELL RECEPTOR SIGNALING PATHWAY
KEGG HUNTINGTONS DISEASE
−6
−4
−2
0
p.adjust
0.003
0.002
0.001

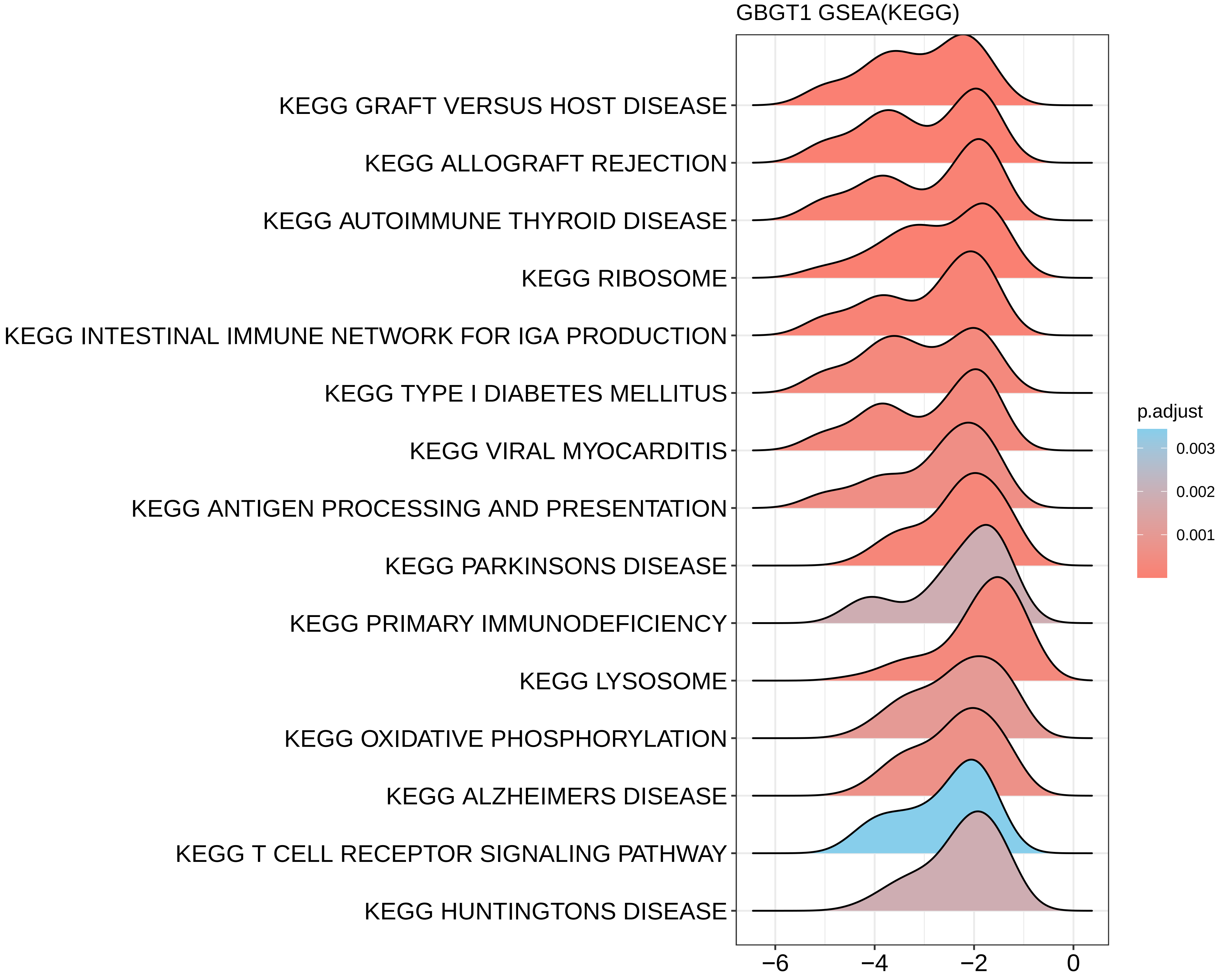
GBGT1 GSEA(KEGG)
KEGG GRAFT VERSUS HOST DISEASE
KEGG ALLOGRAFT REJECTION
KEGG AUTOIMMUNE THYROID DISEASE
KEGG RIBOSOME
KEGG INTESTINAL IMMUNE NETWORK FOR IGA PRODUCTION
KEGG TYPE I DIABETES MELLITUS
KEGG VIRAL MYOCARDITIS
KEGG ANTIGEN PROCESSING AND PRESENTATION
KEGG PARKINSONS DISEASE
KEGG PRIMARY IMMUNODEFICIENCY
KEGG LYSOSOME
KEGG OXIDATIVE PHOSPHORYLATION
KEGG ALZHEIMERS DISEASE
KEGG T CELL RECEPTOR SIGNALING PATHWAY
KEGG HUNTINGTONS DISEASE
−6
−4
−2
0
p.adjust
0.003
0.002
0.001

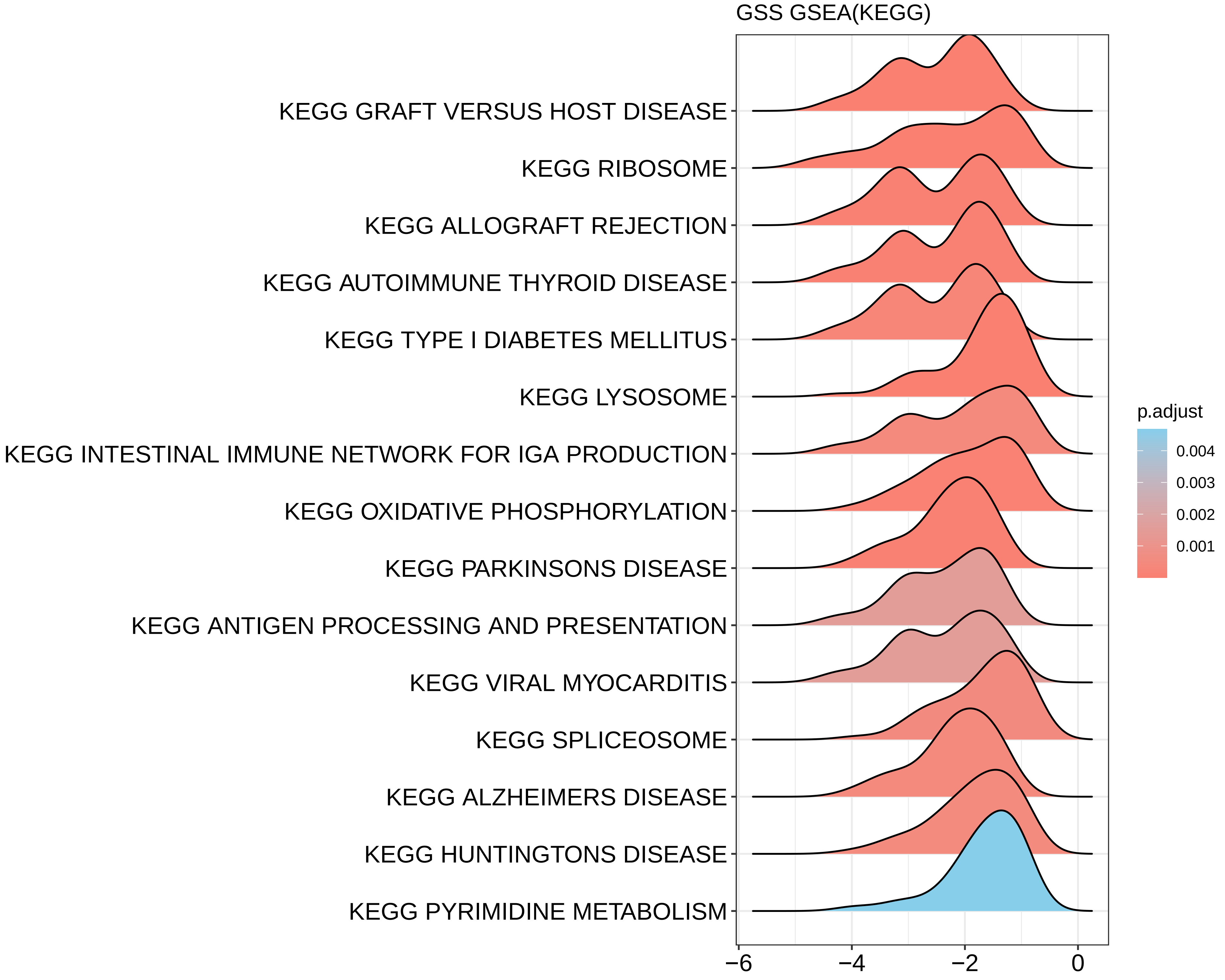

GSS GSEA(KEGG)
KEGG GRAFT VERSUS HOST DISEASE
KEGG RIBOSOME
KEGG ALLOGRAFT REJECTION
KEGG AUTOIMMUNE THYROID DISEASE
KEGG TYPE I DIABETES MELLITUS
KEGG LYSOSOME
KEGG INTESTINAL IMMUNE NETWORK FOR IGA PRODUCTION
KEGG OXIDATIVE PHOSPHORYLATION
KEGG PARKINSONS DISEASE
KEGG ANTIGEN PROCESSING AND PRESENTATION
KEGG VIRAL MYOCARDITIS
KEGG SPLICEOSOME
KEGG ALZHEIMERS DISEASE
KEGG HUNTINGTONS DISEASE
KEGG PYRIMIDINE METABOLISM
−6
−4
−2
0
p.adjust
0.004
0.003
0.002
0.001

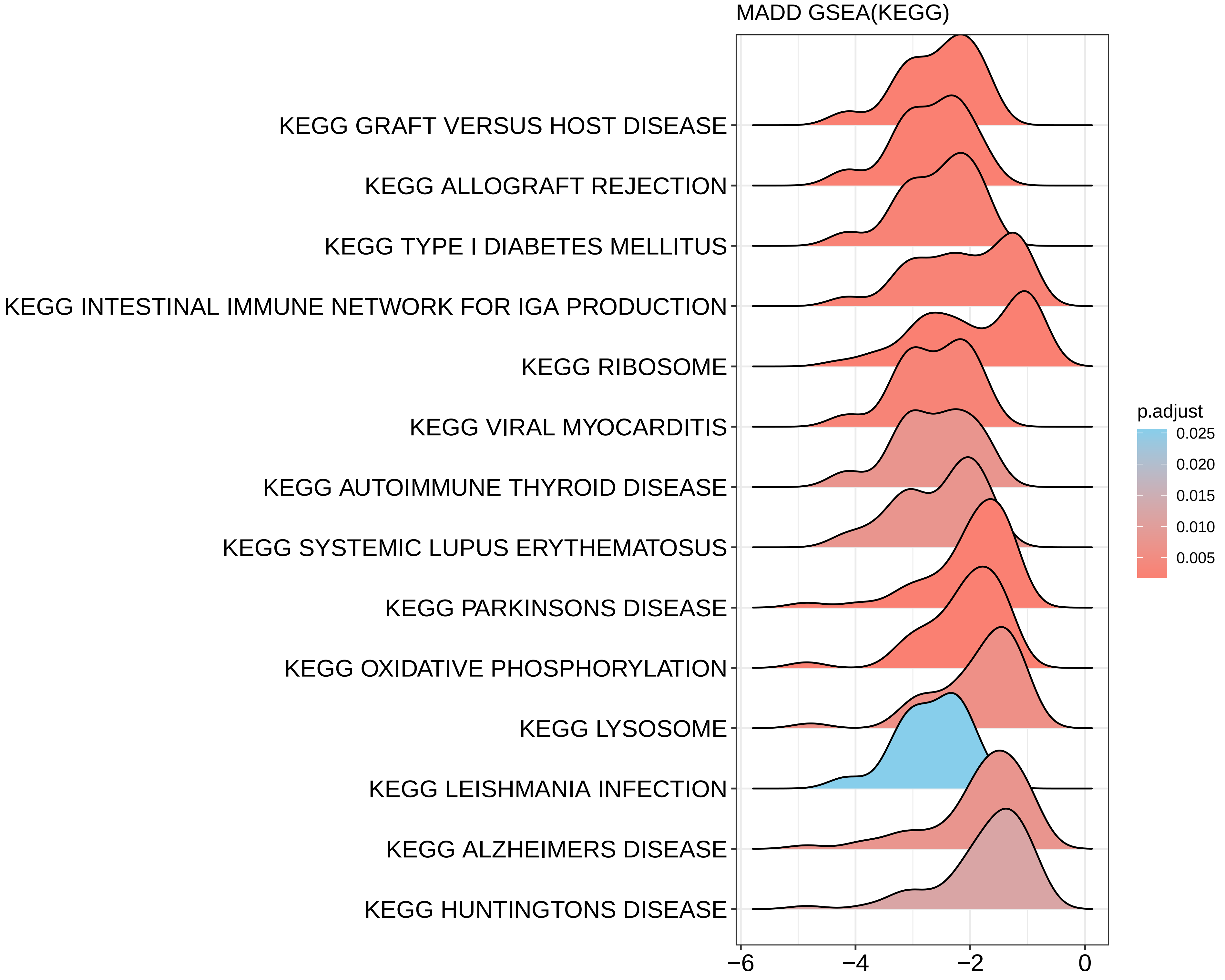

MADD GSEA(KEGG)
KEGG GRAFT VERSUS HOST DISEASE
KEGG ALLOGRAFT REJECTION
KEGG TYPE I DIABETES MELLITUS
KEGG INTESTINAL IMMUNE NETWORK FOR IGA PRODUCTION
KEGG RIBOSOME
KEGG VIRAL MYOCARDITIS
KEGG AUTOIMMUNE THYROID DISEASE
KEGG SYSTEMIC LUPUS ERYTHEMATOSUS
KEGG PARKINSONS DISEASE
KEGG OXIDATIVE PHOSPHORYLATION
KEGG LYSOSOME
KEGG LEISHMANIA INFECTION
KEGG ALZHEIMERS DISEASE
KEGG HUNTINGTONS DISEASE
−6
−4
−2
0
p.adjust
0.025
0.020
0.015
0.010
0.005

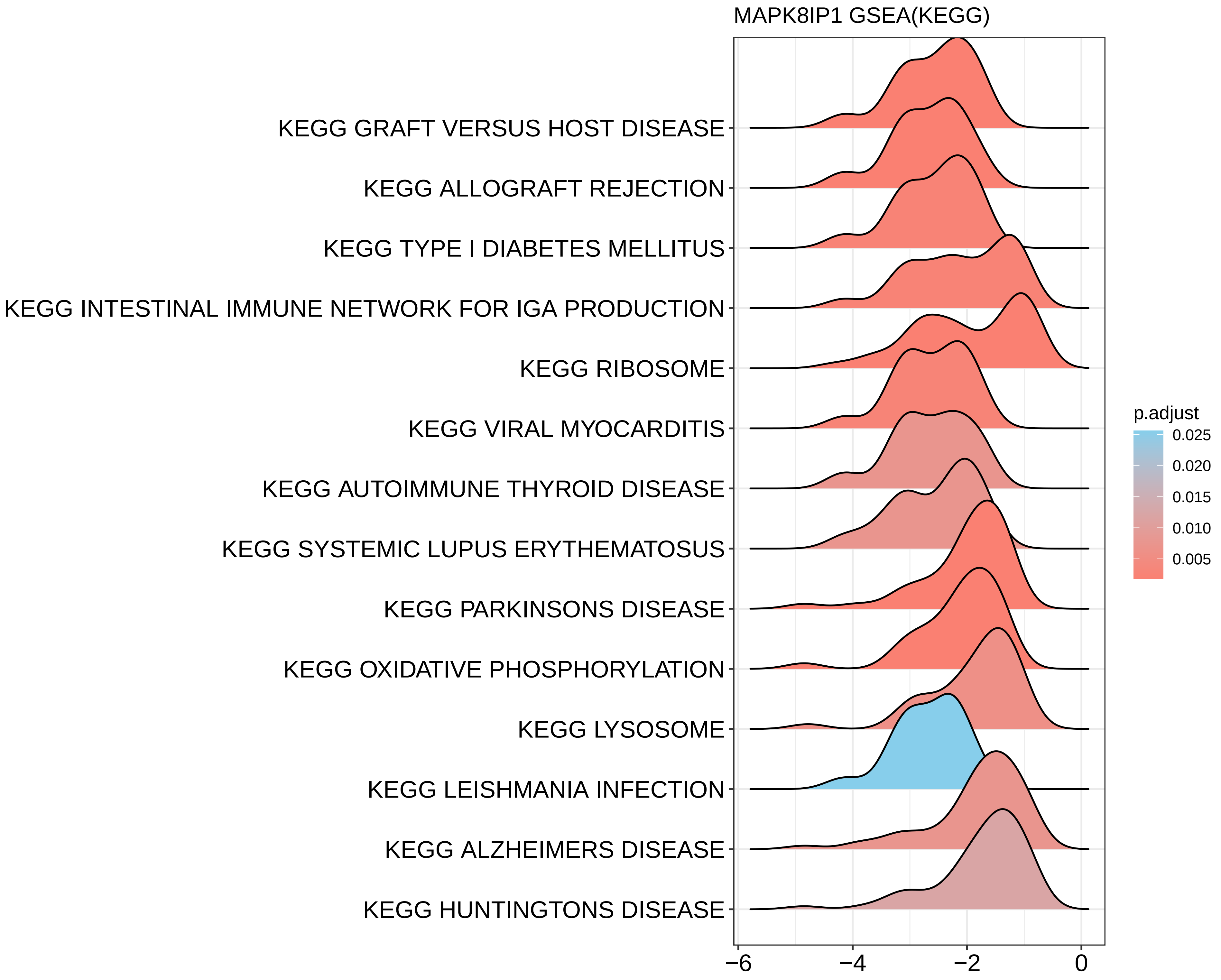

MAPK8IP1 GSEA(KEGG)
KEGG GRAFT VERSUS HOST DISEASE
KEGG ALLOGRAFT REJECTION
KEGG TYPE I DIABETES MELLITUS
KEGG INTESTINAL IMMUNE NETWORK FOR IGA PRODUCTION
KEGG RIBOSOME
KEGG VIRAL MYOCARDITIS
KEGG AUTOIMMUNE THYROID DISEASE
KEGG SYSTEMIC LUPUS ERYTHEMATOSUS
KEGG PARKINSONS DISEASE
KEGG OXIDATIVE PHOSPHORYLATION
KEGG LYSOSOME
KEGG LEISHMANIA INFECTION
KEGG ALZHEIMERS DISEASE
KEGG HUNTINGTONS DISEASE
−6
−4
−2
0
p.adjust
0.025
0.020
0.015
0.010
0.005

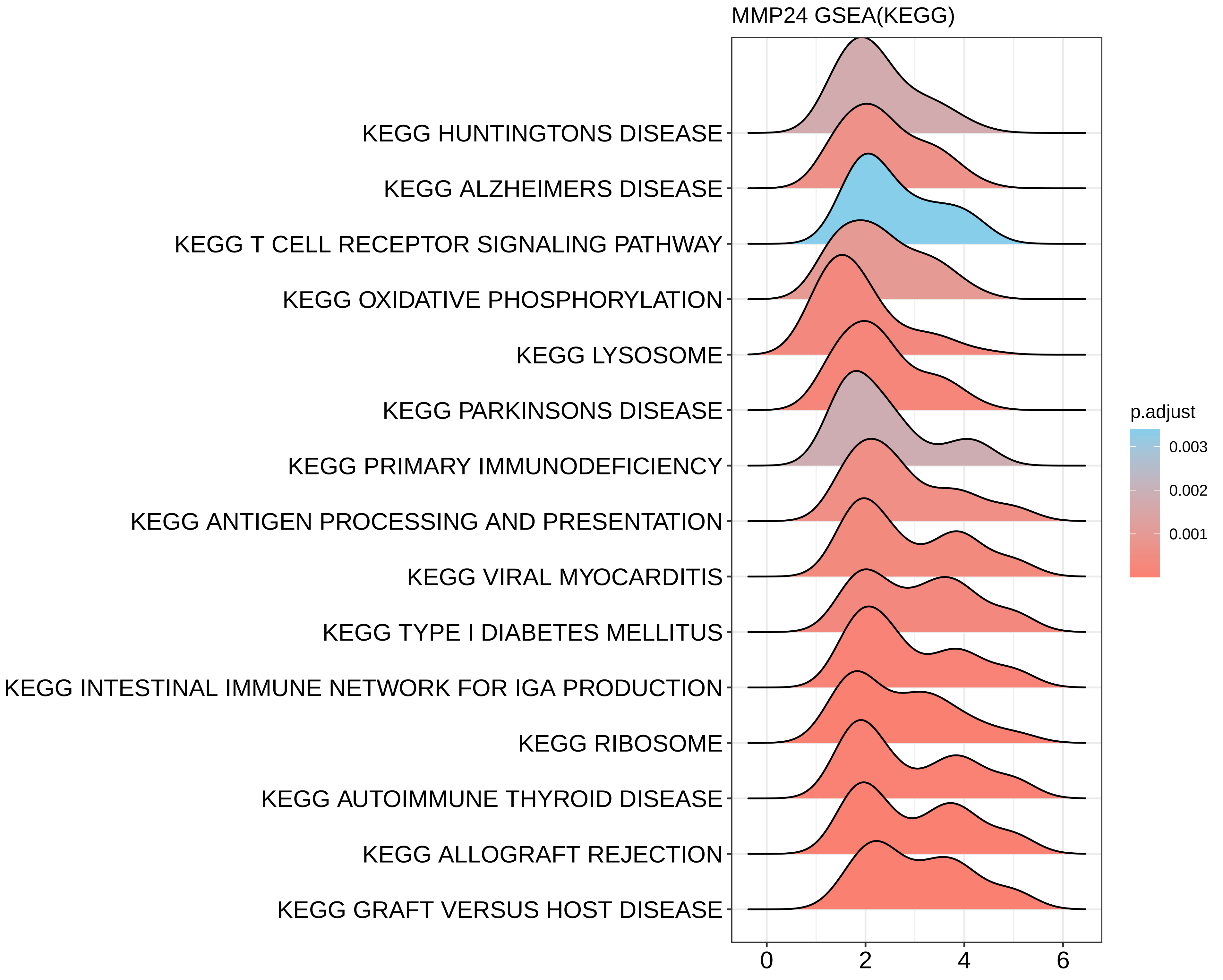

MMP24 GSEA(KEGG)
KEGG HUNTINGTONS DISEASE
KEGG ALZHEIMERS DISEASE
KEGG T CELL RECEPTOR SIGNALING PATHWAY
KEGG OXIDATIVE PHOSPHORYLATION
KEGG LYSOSOME
KEGG PARKINSONS DISEASE
KEGG PRIMARY IMMUNODEFICIENCY
KEGG ANTIGEN PROCESSING AND PRESENTATION
KEGG VIRAL MYOCARDITIS
KEGG TYPE I DIABETES MELLITUS
KEGG INTESTINAL IMMUNE NETWORK FOR IGA PRODUCTION
KEGG RIBOSOME
KEGG AUTOIMMUNE THYROID DISEASE
KEGG ALLOGRAFT REJECTION
KEGG GRAFT VERSUS HOST DISEASE
0
2
4
6
p.adjust
0.003
0.002
0.001

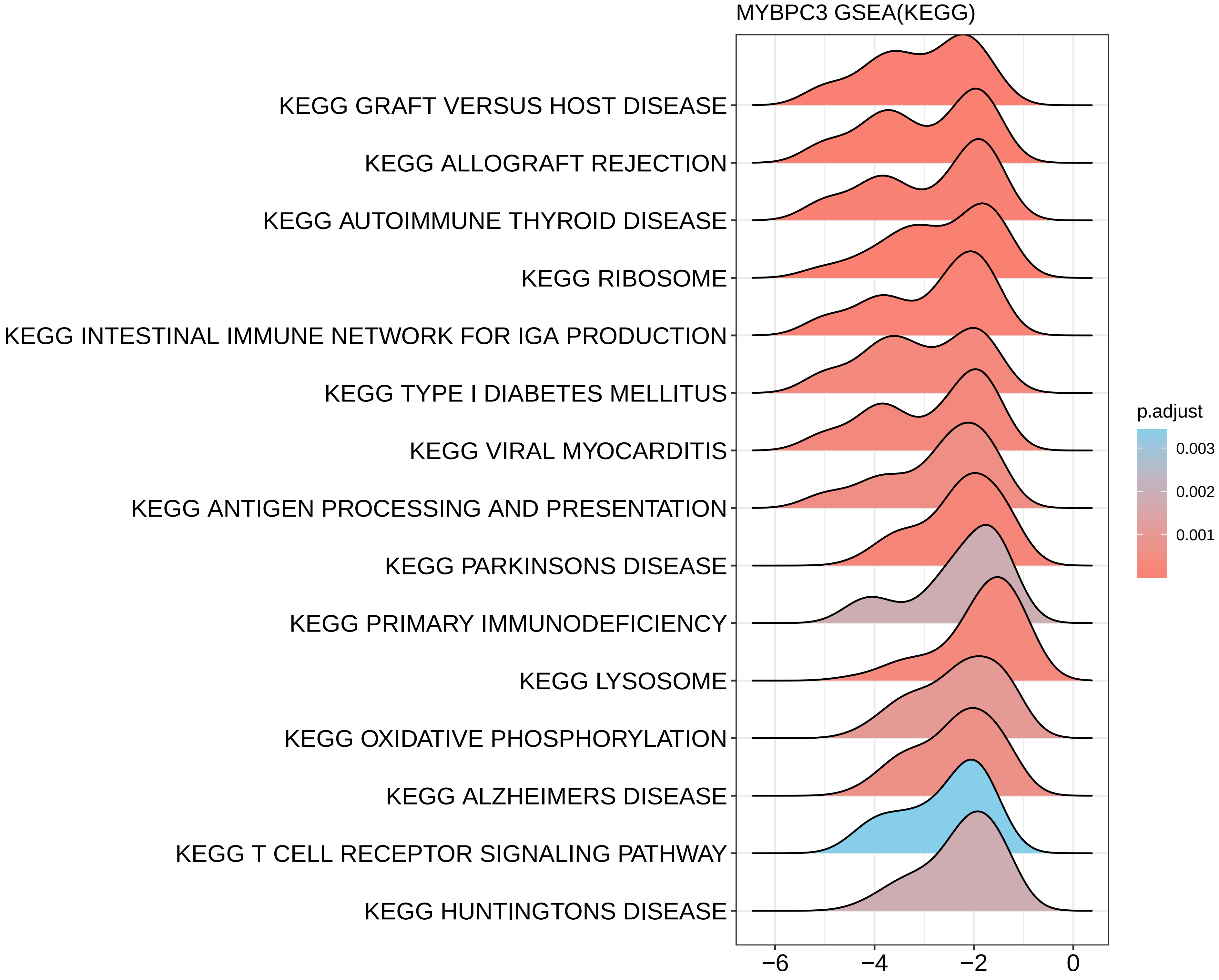

MYBPC3 GSEA(KEGG)
KEGG GRAFT VERSUS HOST DISEASE
KEGG ALLOGRAFT REJECTION
KEGG AUTOIMMUNE THYROID DISEASE
KEGG RIBOSOME
KEGG INTESTINAL IMMUNE NETWORK FOR IGA PRODUCTION
KEGG TYPE I DIABETES MELLITUS
KEGG VIRAL MYOCARDITIS
KEGG ANTIGEN PROCESSING AND PRESENTATION
KEGG PARKINSONS DISEASE
KEGG PRIMARY IMMUNODEFICIENCY
KEGG LYSOSOME
KEGG OXIDATIVE PHOSPHORYLATION
KEGG ALZHEIMERS DISEASE
KEGG T CELL RECEPTOR SIGNALING PATHWAY
KEGG HUNTINGTONS DISEASE
−6
−4
−2
0
p.adjust
0.003
0.002
0.001

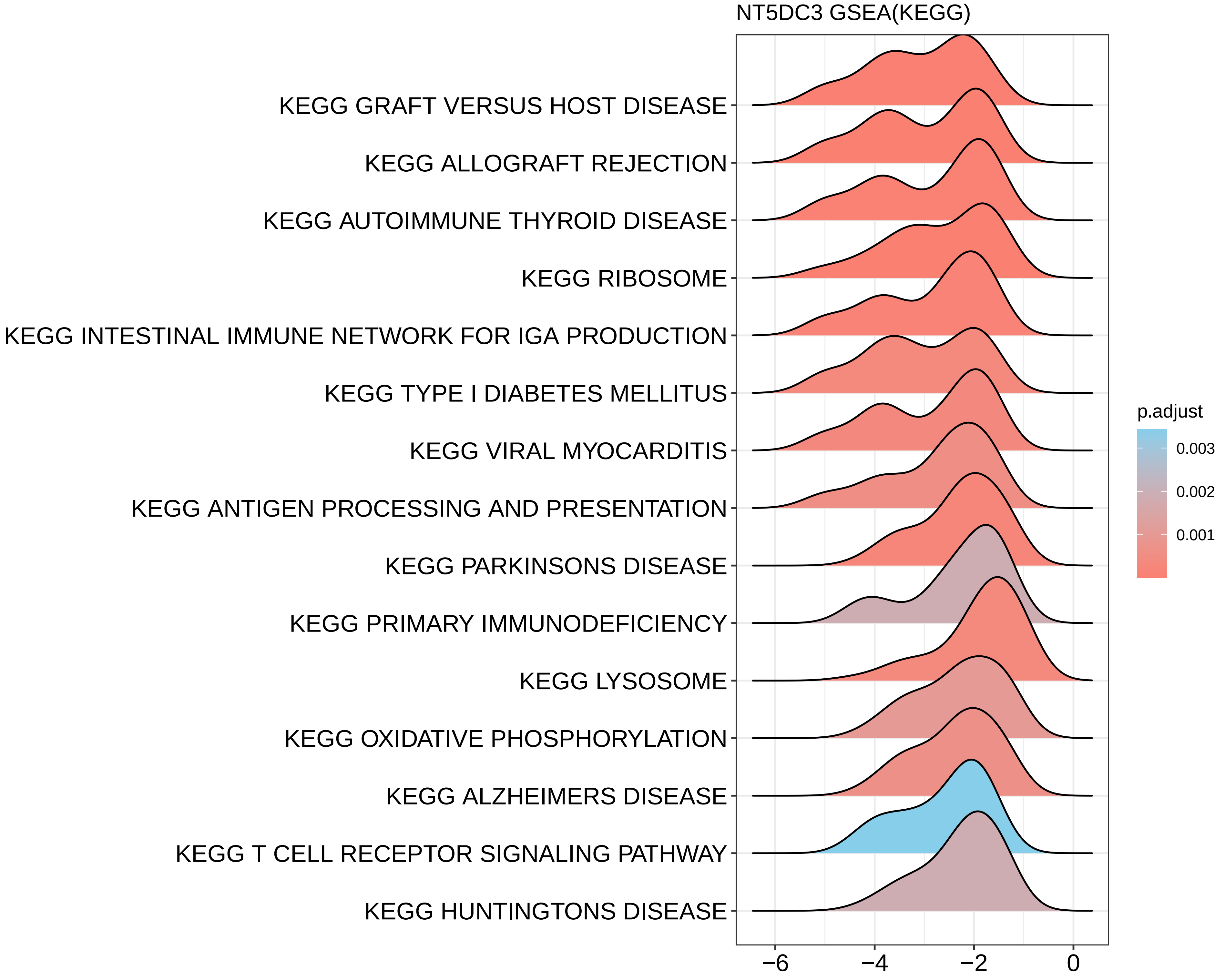

NT5DC3 GSEA(KEGG)
KEGG GRAFT VERSUS HOST DISEASE
KEGG ALLOGRAFT REJECTION
KEGG AUTOIMMUNE THYROID DISEASE
KEGG RIBOSOME
KEGG INTESTINAL IMMUNE NETWORK FOR IGA PRODUCTION
KEGG TYPE I DIABETES MELLITUS
KEGG VIRAL MYOCARDITIS
KEGG ANTIGEN PROCESSING AND PRESENTATION
KEGG PARKINSONS DISEASE
KEGG PRIMARY IMMUNODEFICIENCY
KEGG LYSOSOME
KEGG OXIDATIVE PHOSPHORYLATION
KEGG ALZHEIMERS DISEASE
KEGG T CELL RECEPTOR SIGNALING PATHWAY
KEGG HUNTINGTONS DISEASE
−6
−4
−2
0
p.adjust
0.003
0.002
0.001

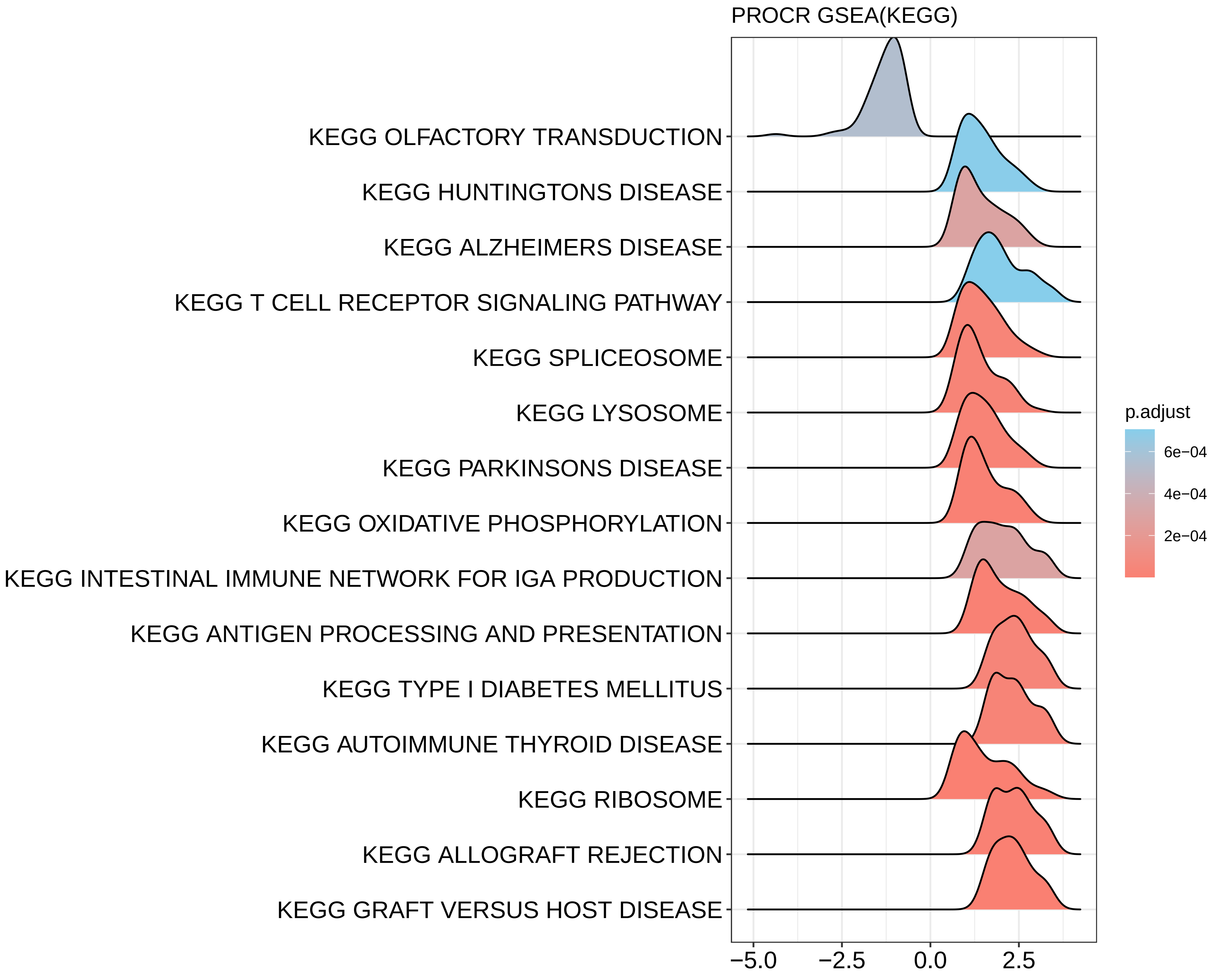

PROCR GSEA(KEGG)
KEGG OLFACTORY TRANSDUCTION
KEGG HUNTINGTONS DISEASE
KEGG ALZHEIMERS DISEASE
KEGG T CELL RECEPTOR SIGNALING PATHWAY
KEGG SPLICEOSOME
KEGG LYSOSOME
KEGG PARKINSONS DISEASE
KEGG OXIDATIVE PHOSPHORYLATION
KEGG INTESTINAL IMMUNE NETWORK FOR IGA PRODUCTION
KEGG ANTIGEN PROCESSING AND PRESENTATION
KEGG TYPE I DIABETES MELLITUS
KEGG AUTOIMMUNE THYROID DISEASE
KEGG RIBOSOME
KEGG ALLOGRAFT REJECTION
KEGG GRAFT VERSUS HOST DISEASE
−5.0
−2.5
0.0
2.5
p.adjust
6e−04
4e−04
2e−04

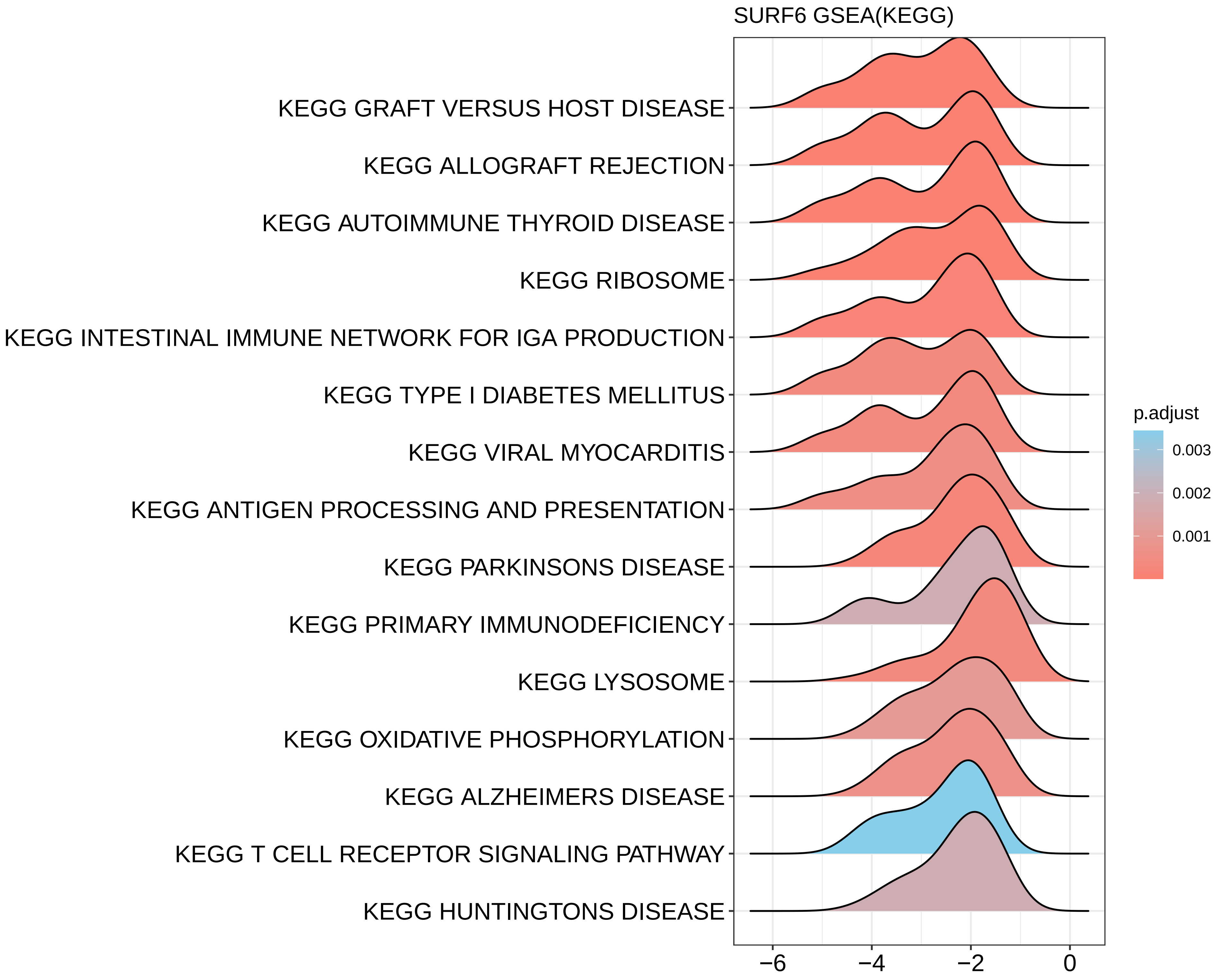

SURF6 GSEA(KEGG)
KEGG GRAFT VERSUS HOST DISEASE
KEGG ALLOGRAFT REJECTION
KEGG AUTOIMMUNE THYROID DISEASE
KEGG RIBOSOME
KEGG INTESTINAL IMMUNE NETWORK FOR IGA PRODUCTION
KEGG TYPE I DIABETES MELLITUS
KEGG VIRAL MYOCARDITIS
KEGG ANTIGEN PROCESSING AND PRESENTATION
KEGG PARKINSONS DISEASE
KEGG PRIMARY IMMUNODEFICIENCY
KEGG LYSOSOME
KEGG OXIDATIVE PHOSPHORYLATION
KEGG ALZHEIMERS DISEASE
KEGG T CELL RECEPTOR SIGNALING PATHWAY
KEGG HUNTINGTONS DISEASE
−6
−4
−2
0
p.adjust
0.003
0.002
0.001

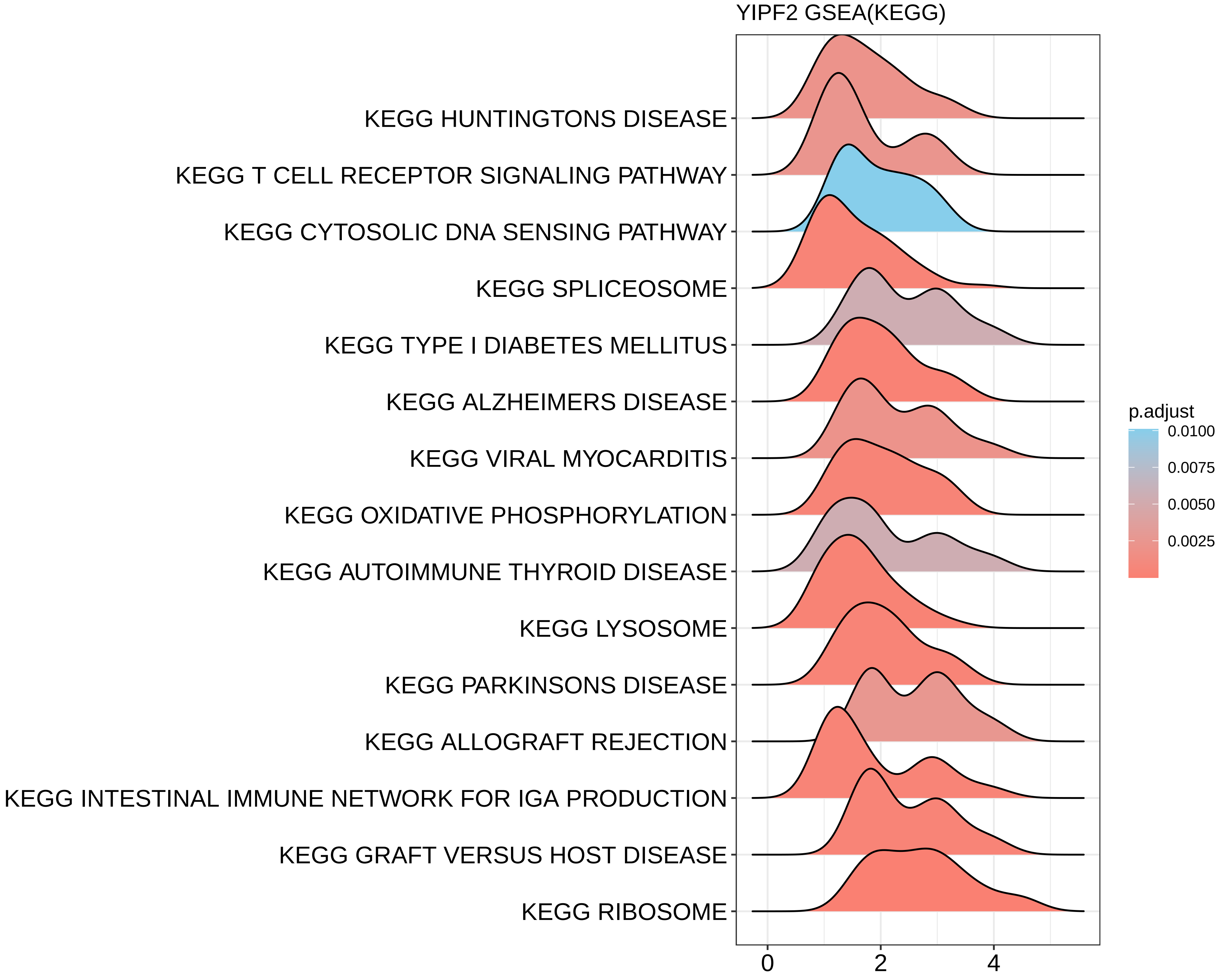

YIPF2 GSEA(KEGG)
KEGG HUNTINGTONS DISEASE
KEGG T CELL RECEPTOR SIGNALING PATHWAY
KEGG CYTOSOLIC DNA SENSING PATHWAY
KEGG SPLICEOSOME
KEGG TYPE I DIABETES MELLITUS
KEGG ALZHEIMERS DISEASE
KEGG VIRAL MYOCARDITIS
KEGG OXIDATIVE PHOSPHORYLATION
KEGG AUTOIMMUNE THYROID DISEASE
KEGG LYSOSOME
KEGG PARKINSONS DISEASE
KEGG ALLOGRAFT REJECTION
KEGG INTESTINAL IMMUNE NETWORK FOR IGA PRODUCTION
KEGG GRAFT VERSUS HOST DISEASE
KEGG RIBOSOME
0
2
4
p.adjust
0.0100
0.0075
0.0050
0.0025
